# Veterinary Medical Association of New Jersey

**Published:** 1890-09

**Authors:** W. H. Cooper

**Affiliations:** Secretary


*Veterinary Medical Association of New Jersey.*—The New Jersey Veterinary Medical Association held one of its best and largest meetings at the Coleman House, Asbury Park, on Thursday, August 14th, with President Dr. J. W. Hawk, in the chair. About twenty-five members and a number of visitors were present from different parts of the State, with delegates from other States. Dr. Hawk made an address on the conduct of veterinarians throughout the State, and the good standing, both financial and professional, of the association. Dr. R. R. Letts read a paper on "Parturient Apoplexy," and a lively discussion followed from a number of members. Dr. Frank Herbert, of Marboro, and J. M. Everett, of Hackettstown, were admitted as active members. Dr. W. W. Curry was appointed the next essayist. A clipping was read from a report of Dr. E. M. Hunt, Secretary of the State Board of Health, in which he states that there is scarcely any pleuro-pneumonia among the cattle of this State, the disease being thoroughly mastered; also, that there are still some cases of tuberculosis, but that disease is not prevailing in any alarming extent. The Association differed with Dr. Hunt, and state that in certain localities of the State the disease does prevail to an alarming extent. The meeting adjourned to meet again in December at Boundbrook. A feature was a handsome dinner, with floral decorations, provided by Mr. Ralph, of the Coleman.

W. H. Cooper, *Secretary.*

